# Scar carcinoma – a real entity or a historical ‘histological curiosity’?

**DOI:** 10.7196/AJTCCM.2020.v26i1.066

**Published:** 2020-03-19

**Authors:** Ismail S Kalla

**Affiliations:** Division of Pulmonology and Critical Care Medicine, Charlotte Maxeke Johannesburg Academic Hospital, University of the Witwatersrand, Johannesburg, South Africa

## Editorial

In this edition of the AJTCCM, we are reintroduced to the concept of ‘scarcinoma’, referring to a carcinoma that originates from peripheral scarring of lung tissue. The first real description of this entity was by the histopathologist Friederich^[1]^ in 1939, but it is not included as a distinct histological subtype in the latest International Association for Lung Cancer study.^[1,2]^ Brett *et al*. ^[3]^ reviewed radiological data from ~900 patients over a 5-year period with a histologically confirmed diagnosis of lung cancer, and describe the presence of scarring within the lungs present on computed tomography (CT) of the chest.^[3]^ Their findings demonstrate a clear association, with one-third of the patients reviewed having scar tissue present within the lungs within the same lobe as the diagnosed cancer.

This is an important study in that it examines the largest cohort to date describing ‘scarcinoma’ in a study population with a high prevalence of smoking and one of the highest burdens of pulmonary tuberculosis (TB) universally.^[4]^ Most of the debate around the entity of scar-carcinoma hinges on the premise that the scar in the region of the lung cancer is the host response to the malignancy (desmoplastic reaction).^[5,6]^ However, there is the possibility that the inflammatory milieu within the scar actually predisposes the host to the development of a neoplastic process. Patients with idiopathic pulmonary fibrosis (IPF) and other fibrotic lung diseases have increased rates of lung cancer, with a reported prevalence as high as 48% in autopsy series of patients with usual interstitial pneumonia. Anatomically, the distribution of lung cancers in patients with IPF is predominantly lower lobe and peripheral, the same regions in which fibrosis is accentuated in IPF.^[7]^ That this might be a ‘cause and effect’ phenomenon will have to be investigated further. Nonetheless, Brett *et al*. ^[3]^ have documented a similar finding, with the presence of scarring not just in the vicinity of the cancer, but on the CT scan as a whole. They have been able to show that while malignancy and fibrosis are much more common within the same lobe, there is an association with the presence of lung scarring documented anywhere in the lung, and lung cancer.

However, what this study highlights is that in a population with a high prevalence of lung scarring due to chronic ongoing inflammation from undiagnosed as well as untreated infections such as TB, as treating physicians, we have to be vigilant to the presence of an occult malignancy. There is a published body of literature confirming the independent risk factor that pulmonary TB poses for the development of lung cancer.^[5]^ This study adds further support to the recently published lung cancer screening guidelines of the South African Thoracic Society, advocating the use of low-dose CT chest scans as an appropriate tool for identifying lung cancer at a stage when a curative therapeutic option could still be offered to patients. In patients with lung scarring identified on plain chest X-ray, the morphological characteristics of a malignancy can often be hidden by the radiological ‘noise’ associated with fibrosis and anatomical distortion of the lung parenchyma. Therefore, a CT scan of the chest may be of value in identifying malignancies hiding with the scar tissue.^[8]^

Patients with significant risk factors suggestive of ‘scarcinoma’, namely previously documented lung scarring or old TB scarring, a family history suggestive of lung malignancies, a smoking history or an occupational history in the presence of unexplained weight loss and a chronic cough, with or without haemoptysis, need to be investigated further. The diagnostic tool of choice is debatable, but the use of biological imaging such as a positron emission tomography scan has added to our armamentarium in identifying the presence of a ‘scarcinoma’. Furthermore, this study opens the pathway to further investigate the neglected entity of ‘scarcinoma’. With the advent of more specific radiological tools such as magnetic resonance imaging of the lung, and better molecular and immunohistochemical diagnostic markers, we might be able to resolve the debate about whether ‘scarcinoma’ actually exists as a separate clinical entity with a different behavioural pattern.^[9]^
